# Validation of the TLICS and AOSpine injury score for surgical management of paediatric traumatic spinal injuries

**DOI:** 10.1007/s00402-022-04413-5

**Published:** 2022-03-29

**Authors:** Friederike Schömig, Nima Taheri, Hussein Kalaf, Maximilian Muellner, Luis Becker, Matthias Pumberger

**Affiliations:** Center for Musculoskeletal Surgery, Charité – Universitätsmedizin Berlin, Charitéplatz 1, 10117 Berlin, Germany

**Keywords:** Thoracolumbar fracture, Paediatric trauma, Polytrauma, Spine surgery, AOSpine, TLICS

## Abstract

**Introduction:**

Fractures of the thoracolumbar spine in children are rare. Consequently, classification systems providing detailed treatment recommendations as already established in adults are still lacking in the paediatric population. We aimed to evaluate the validity and reliability of the thoracolumbar injury classification and severity score system (TLICS) and the AOSpine injury score in paediatric patients presenting with a traumatic fracture of the thoracolumbar spine.

**Materials and methods:**

Patients younger than 18 years presenting with a traumatic thoracolumbar fracture at a large academic trauma centre between 2010 and 2020 were included retrospectively. Demographic and clinical data were retrieved from electronic medical reports. The AOSpine injury score and TLICS were calculated using plain radiography, magnetic resonance imaging, and/or computed tomography.

**Results:**

Sixty patients with 167 fractures were included. Surgical treatment was performed in 14 patients. The mean AOSpine injury score was 1.49 ± 2.0, the mean TLICS was 1.32 ± 1.65. A significant correlation between the classification systems was found (Spearman *r* = 0.975, *p* < 0.001). Interrater reliability analysis revealed Kappa values of 0.868 for the TLICS and 0.860 for the AOSpine injury score (*p* < 0.001). Contingency table analysis showed a sensitivity of 1.00 and specificity of 0.94 for the AOSpine injury score and a sensitivity of 0.90 and specificity of 0.90 for the TLICS in predicting the performed treatment.

**Conclusions:**

Our results confirm that the TLICS is a valid classification system for determining treatment decisions in paediatric patients and show slightly higher accuracy of the AOSpine injury score as well as high interrater reliabilities for both classification systems.

## Introduction

With an estimated 0.6–0.9% of all spinal trauma cases, paediatric fractures of the thoracolumbar spine are rare. Due to a greater elasticity and compressibility of the bone in general and especially the paediatric spine, trauma is less likely to result in a fracture in children than in adults [1]. This rareness of occurrence is why a systematic approach to these fractures is still missing. Conservative treatment options include observation or corset therapy. The decision for surgical treatment depends not only on factors such as fracture stability, displacement, and neurological status but also on the possible consequences of such fractures on the growing spine [2, 3]. If these spinal injuries are not treated adequately, however, irreversible spinal deformities and sagittal imbalance can occur [3].

In adults, two widely accepted thoracolumbar injury classification systems have been recently established, both of which provide detailed treatment recommendations. The thoracolumbar injury classification and severity score system (TLICS) is based on the injury mechanism, the integrity of the posterior ligamentous complex (PLC), and neurological status. The AOSpine injury score is based on the AOSpine thoracolumbar spine injury classification which takes into account the Magerl scale to describe the fracture morphology and ligamentous complex as well as the neurological status [4–6]. While the two classification systems have been developed for the adult population, they have recently started to be used in the paediatric population as well [7]. While the TLICS has been validated in the paediatric population, there are only a few studies in small patient cohorts comparing it to the AOSpine thoracolumbar spine injury classification [8]. Furthermore, in the existing literature, conflicting results with some studies finding superiority of the TLICS over the AOSpine injury score and others finding a high correlation between the two systems are presented [2, 9].

Ideally, a classification system needs to enable clinicians to distinguish between stable and unstable injuries and guide treatment decisions. Thus, detailed morphologic descriptions are necessary as well as a scoring system correlating the injury’s severity with the need for surgical stabilization [6].

Our study’s aim was to evaluate the validity and reliability of both the TLICS and the AOSpine injury score in paediatric patients presenting at a large academic trauma centre with a traumatic fracture of the thoracolumbar spine.

## Materials and methods

The study was approved by the institutional ethics committee (EA2/046/21), and informed consent was waived. We retrospectively included patients younger than 18 years who presented at our large academic trauma centre with a traumatic fracture of the thoracolumbar spine between January 2010 and December 2020. Exclusion criteria included isolated cervical fractures, nontraumatic or pathological fractures and incomplete clinical or imaging records. Demographic and clinical data such as age, sex, injury mechanism, injury severity scores (ISS), fracture level, surgical treatment, and length of hospital stay were retrieved from electronic medical reports and patient charts. Polytrauma was defined as an ISS of 16 or higher [10, 11]. Surgical management was performed in cases of an injury of the PLC, posterior wall recession with an intracanal fragment, neurological deficit, or vertebral deformities that may cause kyphosis with conservative treatment alone. PLC injury was defined as diastasis of the facet joints on computed tomography (CT) or posterior oedema in the region of PLC elements on T2 short tau inversion recovery (STIR) sagittal magnetic resonance imaging (MRI) [12].

### Image analysis

The AOSpine injury score and TLICS were calculated using plain radiography, magnetic resonance imaging, and/or computed tomography. All available radiological images were analysed by a spine surgeon with eleven years of experience, and a research fellow trained in musculoskeletal radiology with two years of experience. Disagreement was solved in a consensus meeting with an orthopaedic surgery resident with three years of experience.

### TLICS

For calculation of the TLICS, the fracture morphology was classified as compression, burst, translation, or distraction injury. The integrity of the PLC was graded as intact, suspected injury, or injured and the neurological status was graded as intact, nerve root involvement, complete neurologic or conus medullaris injury or incomplete neurologic or conus medullaris injury. The assignment of points for the TLICS is presented in Table 1 [13, 14].

**Table 1:** TLICS

	Assigned points
**Fracture morphology**	
Compression	1
Burst	2
Translation/rotation	3
Distraction	4
**Integrity of the posterior ligamentous complex**	
Intact	0
Injury suspected/indeterminate	2
Injured	3
**Neurological status**	
Nerve root	2
Cord, conus medullaris incomplete	3
Cord, conus medullaris complete	2
Cauda equina	3

With three or fewer points, conservative treatment is recommended, with four points, either conservative or surgical treatment may be performed; with five points or more, surgical treatment is recommended [13, 14]

### AOSpine injury score

The AOSpine injury score was calculated based on the AOSpine thoracolumbar classification system taking into account the injury morphology according to the Magerl scale and neurological status. Injury morphology was classified as an A (compression), B (distraction), or C (translation) type injury. Type A fractures were graded as A0 (process fracture), A1 (compression/wedge), A2 (split/pincer), A3 (burst involving one endplate), or A4 (burst involving both endplates). Type B fractures were graded as B1 (bony Chance fracture), B2 (failure of the posterior tension band), or B3 (hyperextension injury). The neurological status was classified as N0 (intact), N1 (resolved transient injury), N2 (radiculopathy), N3 (incomplete spinal cord/cauda equina), N4 (complete spinal cord), or Nx (indeterminable). The assignment of points for the AOSpine injury score is presented in Table 2. Surgical treatment is recommended for an AOSpine injury score of five points or more while in patients with four or five points treatment may be conservative or surgical [6].

**Table 2:** AOSpine injury score

	Assigned points
**Magerl classification**	
A0: process fracture	0
A1: compression/wedge	1
A2: split/pincer type	2
A3: incomplete burst	3
A4: complete burst	5
B1: posterior transosseous disruption	5
B2: posterior ligamentous disruption	6
B3: anterior ligamentous distruption	7
C: translation	8
**Neurological status**	
N0: intact	0
N1: transient deficit	1
N2: radiculopathy	2
N3: incomplete spinal cord injury	4
N4: complete spinal cord injury	4

With less than four points, conservative treatment is recommended; with four or five points, either conservative or surgical treatment may be performed; with more than five points, surgical treatment is recommended [6]

### Statistical analysis

Descriptive summaries were calculated as the means with standard deviation. To determine interrater reliability, Cohen’s Kappa was calculated. Kappa values of < 0.00 were rated as poor, 0.00–0.20 as slight, 0.21–0.40 as fair, 0.41–0.60 as moderate, 0.61–0.80 as substantial, and 0.81–1.00 as almost perfect agreement [15]. The convergence of the AOSpine classification score to the TLICS score was analysed using Spearman rank correlation analysis. Contingency table analysis was performed to calculate the scores’ accuracy in predicting the performed treatment. For all tests, a *p* value of < 0.05 was considered significant. SPSS version 27 (SPSS Inc., Chicago, Illinois) was used for statistical analysis.

## Results

Ninety-eight patients younger than 18 years presented at our trauma centre with a spine fracture. Twelve patients were excluded due to isolated cervical fractures, eight were excluded because they presented with pathological fractures as a result of bone or oncologic diseases and 18 were excluded due to incomplete clinical or imaging data yielding a total of 60 patients with 167 fractures included in our analysis.

Patient characteristics are listed in Table 3. Thirty-two male (53.3%) and 28 female (46.7%) patients with a mean age of 13.13 ± 3.93 years were included. The most common trauma mechanism was fallen (73.3%) followed by traffic accidents (23.3%) and sports injuries (3.3%). The mean ISS was 22.62 ± 19.46. Thirty-four patients had an ISS > 15 and were therefore classified as having polytrauma. The most common concomitant injuries were head injuries (46.7%) followed by thoracic (38.3%) and abdominal (31.7%) injuries. Fractures of the thoracic spine (51.5%) were slightly more common than fractures of the lumbar spine (48.5%). A mean of 2.97 ± 2.31 spinal levels per patient was affected. Plain radiography was performed in 59 (98.3%), CT in 44 (73.3%), and MRI in 28 (46.7%) patients. Surgical treatment was performed in 14 patients (23.3%).

**Table 3:** Demographic data of the included patients

Age (years)	13.13 ± 3.93
Sex (m:w)	32:28
Trauma mechanism	
Fall	44 (73.3%)
Traffic accident	14 (23.3%)
Sports trauma	2 (3.3%)
Injury Severity Score	22.62 ± 19.46
Polytrauma	34 (56.7%)
Concomitant injuries	
Head	28 (46.7%)
Thorax	23 (38.3%)
Abdomen	19 (31.7%)
Pelvis	14 (23.3%)
Lower extremities	11 (18.3%)
Upper extremities	13 (21.7%)
Symptoms	
Pain	59 (98.3%)
Neurological deficit	2 (3.3%)
Reduced consciousness	16 (26.7%)
Fracture location	
Thoracic	86 (51.5%)
Lumbar	81 (48.5%)
Number of affected levels	2.97 ± 2.31
Treatment	
Conservative	46 (76.7%)
Surgical	14 (23.3%)
Length of hospital stay (days)	11.27 ± 14.89

For the TLICS, Cohen’s Kappa was 0.876 (*p* < 0.001) for fracture morphology, 0.922 (*p* < 0.001) for PLC injury, and 0.868 (*p* < 0.001) for total TLICS. For the AOSpine thoracolumbar classification, Cohen’s Kappa was 0.899 (*p* < 0.001) for fracture morphology and 0.860 (*p* < 0.001) for the total AOSpine injury score.

Utilizing the TLICS, 103 (61.7%) fractures were classified as compression fractures, twelve (7.2%) as burst fractures, 13 (7.8%) as rotation/translation fractures, and one (0.6%) as a distraction fracture. The PLC was intact in 152 (91.0%) fractures and injured in 15 (9.0%) fractures. The neurological status was classified as intact in 58 (96.7%) patients, as nerve root involvement in one (0.6%) patient and as a complete cord injury in another (0.6%) patient. The mean TLICS was 1.32 ± 1.65. The TLICS per patient and associated treatment is shown in Table 4. Contingency table analysis showed a sensitivity of 0.90 and specificity of 0.90 in predicting the performed treatment (Table 5).

**Table 4:** TLICS per patient and performed treatment

TLICS	Performed treatment	
	Conservative	Surgical
0	9	0
1	31	3
2	4	2
3	1	0
5	0	1
6	0	7
7	1	0
9	0	1

**Table 5:** Contingency table analysis of treatment decisions made according to the TLICS

	Proposed surgical	Proposed conservative	Total			
Surgical	9	5	14	SE	0.90	0.70–1.00
Conservative	1	45	46	SP	0.90	0.82–0.98
Total	10	50		PPV	0.64	0.39–0.89
				NPV	0.98	0.94–1.00

Data are given with 95% confidence intervals

*SE* sensitivity, *SP* specificity, *PPV* positive predictive value, *NPV* negative predictive value

According to the AOSpine thoracolumbar classification system, there were 38 (22.8%) A0, 102 (61.1%) A1, one (0.6%) A2, nine (5.4%) A3, three (1.8%) A4, one (1.8%) B1, six (3.6%) B2, and seven (4.2%) C fractures. Fifty (82.0%) patients did not show a preoperative neurological deficit, one (1.6%) case was classified as N1 and another as N4. Nine patients (5.4%) could not be evaluated neurologically. The mean AOSpine injury score was 1.49 ± 2.0. The AOSpine injury score per patient and associated treatment is shown in Table 6. Contingency table analysis showed a sensitivity of 1.00 and specificity of 0.94 in predicting the performed treatment (Table 7).

**Table 6:** AOSpine injury score per patient and performed treatment

AOSpine Injury Score	Performed treatment	
	Conservative	Surgical
0	9	0
1	30	2
2	2	1
3	4	0
5	1	3
6	0	3
8	0	4
10	0	1

**Table 7:** Contingency table analysis of treatment decisions made according to the AOSpine score

	Proposed surgical	Proposed conservative	Total			
Surgical	11	3	14	SE	1.00	1.00–1.00
Conservative	0	45	45	SP	0.94	0.87–1.00
Total	11	48		PPV	0.79	0.58–1.00
				NPV	1.00	1.00–1.00

Data are given with 95% confidence intervals

*SE* sensitivity, *SP* specificity, *PPV* positive predictive value, *NPV* negative predictive value

Spearman correlation analysis showed a significant correlation between the TLICS and the AOSpine injury score (*r* = 0.975, *p* < 0.001).

## Discussion

Paediatric fractures of the thoracolumbar spine are rare which is why validated classification and treatment algorithms are still lacking even though these injuries represent a unique challenge for surgeons. We therefore aimed to analyse the validity and reliability of both the TLICS and the AOSpine injury score in assessing thoracolumbar fractures in children. Our study substantiates convergence of the AOSpine injury score to the TLICS and shows high validity of both scoring systems in predicting the performed treatment of traumatic fractures of the thoracolumbar spine in children.

To date, there is no accepted classification system for the management of paediatric spinal fractures. Both the AOSpine thoracolumbar classification system and the TLICS have only been validated in adult populations, in which both the AOSpine injury score and the TLICS show good to excellent reliability and validity [16–18]. There is, however, still a lack of studies analysing these classification systems in children. While there are multiple studies showing good interrater reliability for the TLICS and good validity for predicting operative versus conservative treatment in children [7, 8, 19], it was not until 2019, that Mo et al. showed agreeability and reliability between the AOSpine thoracolumbar classification system, TLICS, and intraoperative findings [2]. A recent study comparing the AOSpine injury score to the TLICS showed a significant correlation of both classification systems with surgical management decisions but found the TLICS to be more appropriate [9].

In line with these previous studies, we showed excellent interrater reliability for both the AOSpine injury score (0.860, *p* < 0.001) and the TLICS (0.868, *p* < 0.001). Furthermore, our results revealed that both the AOSpine injury score (sensitivity 1.00, specificity 0.94) and the TLICS (sensitivity 0.90, specificity 0.90) were valid for predicting conservative versus surgical treatment in the management of paediatric patients. In the three patients who received surgical treatment, even though conservative treatment was indicated according to the AOSpine injury score, the decision for surgical treatment was based on a high degree of spinal canal stenosis caused by a burst fracture, disruption of the costovertebral articulation on the fracture level, or fracture-related kyphosis with the potential for progressive deformity. As previously described, the potential for progressive deformity and severe back pain needs to be taken into account if there is significant kyphosis, which is why a kyphotic angle of over 20° is thought to be indicative of local instability [1, 20]. Similar to the adult population, in these mentioned cases, an individual decision-making is warranted to find the best treatment of choice. In the case of conservative management, close follow-up examinations and clinical reevaluations need to be performed.

Disagreement between the AOSpine injury score and TLICS in the proposed treatment was only seen in A4 burst fractures with no injury of the PLC, which receive fewer points on the TLICS scale than on the AOSpine injury score scale. Overall, we do, however, substantiate convergence from the AOSpine injury score to the TLICS.

As the anatomy and biomechanics of children vary significantly not only from those of adults but also between different paediatric age groups, the injury patterns differ as well. Due to a relatively large head combined with a mostly cartilaginous spine, especially young children show a hypermobile spine which predisposes them to upper spine injuries [21]. With increasing age, ossification of the vertebrae starts, the facets orientate more vertically and the uncinate process protrudes, which in turn predisposes older children to lower spine injuries similar to those in the adult population [22, 23]. Accordingly, in our analysis, we found an increase in injuries to the lumbar spine with increasing age: 24.0% of patients younger than 14 years old showed a lumbar fracture compared to 42.9% in patients older than 14 years old. Therefore, in developing future spinal classification systems specifically for children, the anatomical and biomechanical characteristics of different age groups need to be taken into consideration. Our results do, however, show that regarding the overall paediatric population, both the TLICS and the AOSpine injury score show high validity in predicting the performed treatment.

Some limitations need to be discussed. Our analysis is limited by the small sample size of 60 patients and retrospective data collection. This may have caused our statistical analysis to be underpowered. Furthermore, subgroup analyses of different age groups were not feasible. However, due to the rareness of these injuries, to our knowledge, this is still one of the largest analyses of 167 traumatic paediatric fractures of the thoracolumbar spine. As our study was conducted retrospectively, different imaging modalities were used and treatment decisions were made by different surgeons based on their experience and were therefore not controlled. Furthermore, due to our study’s retrospective design, sufficient follow-up data were not available for our analysis, which is why further prospective studies are needed to substantiate our findings.

In conclusion, our results confirm that both the TLICS and the AOSpine injury score are valid classification systems for determining whether conservative or surgical treatment is indicated for thoracolumbar spinal fractures in the paediatric population. In neurologically intact complete burst fractures with an intact PLC status, the TLICS recommends conservative treatment, which is why we found a slight tendency towards a higher validity of the AOSpine injury score. However, as both classification systems cannot possibly depict every fracture, in certain cases individual treatment decisions need to be made. Furthermore, as the anatomy and biomechanics of children vary significantly, there still is a need for the development of a classification system taking into account the distinct characteristics of different paediatric age groups.

## Data Availability

The datasets generated and/or analyzed during the current study are not publicly available due to patient privacy but are available from the corresponding author on reasonable request.
